# HEALTH-RELATED QUALITY OF LIFE OF OLDER PEOPLE WITH DIABETES DEPENDING ON WHETHER THEY LIVE IN FAMILIES OR NOT

**DOI:** 10.1093/geroni/igac059.2314

**Published:** 2022-12-20

**Authors:** Gi Won Choi, Sun Ju Chang

**Affiliations:** Seoul National University, Seoul, Seoul-t'ukpyolsi, Republic of Korea; Seoul National University, Seoul, Seoul-t'ukpyolsi, Republic of Korea

## Abstract

Diabetes requires self-management, such as the use of insulin, oral antidiabetic drugs, diet, and exercise, for maintaining blood glucose. To promote self-management of older people with diabetes, family support is considered an important factor. However, older people living alone lack family support and have a lower health-related quality of life (HRQOL) than those living in families. The purpose of this study was to assess HRQOL and the factors that affect it between older people with diabetes living alone and those living in families. We performed secondary data analysis using the data of Korea National Health and Nutrition Examination Survey (KNHANES) 2017–2019. In total, 973 people with diabetes aged 65 years or older answered all questions about HRQOL. Complex sample analysis was performed. Each dimension of HRQOL was significantly lower in people living alone than those living in families. Restricted activity, bedridden, number of comorbidities, and perceived health status significantly predicted HRQOL in patients living alone. Health insurance, age, economic activity, unmet medical service needs, restricted activity, bedridden, number of comorbidities, perceived health status, and suicidal ideation significantly predicted HRQOL in patients living in families. This study indicated how differences in HRQOL exist between older patients with diabetes living alone and those living in families. This data will be useful in developing educational programs in the future, and it will be helpful when dealing with people living alone who have diabetes.

